# Perspectives for using genetically encoded fluorescent biosensors in plants

**DOI:** 10.3389/fpls.2013.00234

**Published:** 2013-07-12

**Authors:** Sisse K. Gjetting, Alexander Schulz, Anja T. Fuglsang

**Affiliations:** ^1^Transport Biology Section, Department of Plant and Environmental Sciences, University of CopenhagenCopenhagen, Denmark; ^2^Center of Excellence for Membrane Pumps and Disease, PUMPKINAarhus, Denmark

**Keywords:** plant bioimaging, genetically encoded biosensors, fluorescence microscopy, calcium sensor, phosphatidylinositol sensor, pH sensors

## Abstract

Genetically encoded fluorescent biosensors have long proven to be excellent tools for quantitative live imaging, but sensor applications in plants have been lacking behind those in mammalian systems with respect to the variety of sensors and tissue types used. How can this be improved, and what can be expected for the use of genetically encoded fluorescent biosensors in plants in the future? In this review, we present a table of successful physiological experiments in plant tissue using fluorescent biosensors, and draw some conclusions about the specific challenges plant cell biologists are faced with and some of the ways they have been overcome so far.

## Introduction

Genetically encoded fluorescent biosensors are increasingly used as the preferred method to visualize and analyse ion fluxes, signaling components, and metabolites, covering an expanding palette of cellular processes. While fluorescent proteins as such are mainly used for localization and expression studies, genetically encoded fluorescent biosensors in addition allow real time studies of cell metabolism with a similar high spatial and temporal resolution. Cell-specific promoters allow biosensor expression in the target cell of choice in contrast to chemical probes that are inherently dependent on efficient delivery into the cells.

The huge interest and progress in the field is reflected in a large number of recent reviews on fluorescent proteins and genetically encoded sensors, e.g., (Fehr et al., 2004; Lalonde et al., 2005; VanEngelenburg and Palmer, 2008; Frommer et al., 2009; Chudakov et al., 2010; Okumoto, 2010; Mehta and Zhang, 2011; Miyawaki, 2011; Newman et al., 2011; Palmer et al., 2011; Okumoto et al., 2012). Several reviews describe plant specific uses, e.g., (Dixit et al., 2006; Frommer et al., 2009; Swanson et al., 2011; Choi et al., 2012; Ehrhardt and Frommer, 2012; Okumoto, 2012; Okumoto et al., 2012). In addition, http://biosensor.dpb.carnegiescience.edu/ provides a database of selected available biosensors.

In the present context, the term genetically encoded fluorescent biosensors refers to fluorescent proteins coupled with a sensing mechanism that causes a change in fluorescence intensity upon ligand binding. Most sensors can be grouped within two major types of fluorescent biosensors: (1) single fluorescent protein sensors, which can carry the sensing mechanism within the fluorescent protein, such as e.g., pHluorins, or where sensing is coupled to a ligand binding domain. Other options using single fluorescent proteins include protein-protein interactions reported by fluorescent protein reconstitution (biFC) or detection of protein translocation. One notable exception of specific plant relevance is the DII-Venus auxin sensor, where degradation of the fluorescent protein is utilized as sensing mechanism. (2) FRET-based sensors, where ligand binding causes a conformational change of the sensor leading to a change in FRET ratio between two fluorescent proteins, usually CFP/YFP variants. Within these groups many sensor platform designs are possible, which are described in detail elsewhere, see e.g., (Okumoto et al., 2012).

There is general consensus that the field is expanding and far from saturated with respect to sensor targets, the quality and variety of the fluorescent proteins, spatiotemporal resolution, compartmentation, and to imaging techniques. This paper discusses the perspectives for using genetically encoded fluorescent biosensors in plants, summarizing the specific challenges plant cell biologists are faced with and the ways they have been overcome so far. Although, due to space restrictions, this review focuses on fluorescent biosensors, the aspects discussed apply to luminescent biosensors as well. What are the expectations for fluorescent biosensors in plants in the future? Can they help in assigning a function to the many orphan receptor-like kinases or create complete flux maps of metabolite and ions in Arabidopsis and other model organisms as suggested by (Okumoto et al., 2012)? We will point to some challenges that need to be addressed, if biosensors are to be used more widely.

## Why are genetically encoded fluorescent biosensors less used in plants than in mammalian cells?

The variety of genetically encoded fluorescent sensors is explored primarily in mammalian systems. Although these sensors offer highly attractive advantages for plant cell imaging, reports on physiological measurements in plants have been comparatively few, which might indicate that using these tools for live physiological experiments in plants is not trivial.

Table 1 below is a compilation of genetically encoded fluorescent sensors used for physiological experiments in plants. Several important insights into plant cell biology have been gained from their use. Notably, applications of the Ca^2+^-sensing Cameleons have given substantial insight into the role of calcium in stomatal opening (Allen et al., 1999a,b) as well as the role of calcium gradients in growing pollen tubes (Michard et al., 2008), root hairs of Arabidopsis (Monshausen et al., 2007), Nod factor-induced nuclear calcium transients in *M. truncatula* root hair cells (Capoen et al., 2011) and visualization of Ca^2+^-dynamics in response to auxin during root gravitropism (Monshausen et al., 2011). Also pH sensors have been useful in plants. The pHluorin sensors have been used to document in detail cytosolic pH gradients and oscillations in growing pollen tubes (Michard et al., 2008), and cell wall pH has been measured by use of pHluorins (Gao et al., 2004) or apo-pHusion (Gjetting et al., 2012) secreted to the apoplast.

**Table 1:** **Genetically encoded fluorescent sensors used for physiological experiments in plants, subdivided by sensor function**.

**Sensor name**	**FPs**	**Kd/pKa**	**λ_max_ex./em.**	**Plant species, tissue**	**Localization**	**Stimulus/response**	**References**
**CAMELEONS AND OTHER CALCIUM SENSORS**							
YC2.1	ECFP-EYFP	100 nM, 4.3 μM (biphasic)	ECFP: 434/477 EYFP: 514/427	*A.t*. guard cells	Cytosol	ABA pathogen elicitors CO2	Allen et al., 1999a,b, 2000, 2001, 2002; Hugouvieux et al., 2001; Klusener et al., 2002; Young et al., 2006; Siegel et al., 2009; **Miyawaki et al., 1999**
				*L. longiflorum, N. tabacum,* pollen tubes	Cytosol, nucleus		Watahiki et al., 2004
nupYC2.1				*M. truncatula* root hairs, wt and symbiotic. defect. mutants	Cytosol, nucleus	Nod factor, mycorrhiza, Mastoparan, JA	Miwa et al., 2006a,b; Sun et al., 2007; Kosuta et al., 2008; Sieberer et al., 2009; Capoen et al., 2011
YC3.1	ECFP-EYFP	1.5 μM		*N. tabacum,* pollen tube	Cytosol		Michard et al., 2008, 2011, **Miyawaki et al., 1999**
				*A.t.* pollen grain, pollen tubes, papilla cells	Cytosol		Iwano et al., 2004
YC3.6	ECFP-cpVENUS	250 nM		*A.t.* roots	Cytosol	Glu, ATP, Al^3+^	Haruta et al., 2008; Rincon-Zachary et al., 2010, **Nagai et al., 2004**
				*A.t*., *N. tabacum,* pollen tubes	Cytosol		Iwano et al., 2009
				*A.t.* guard cells	Cytosol	Methyl Jasmonate (MeJA)	Weinl et al., 2008; Munemasa et al., 2011
				*A.t.* rosette leaves	Cytosol	Butyrate, ABA, MeJA	Islam et al., 2010
				*A.t.* roots, root hairs	Cytosol	Touch, gravitropism, barrier, IAA	Monshausen et al., 2007, 2008, 2009, 2011
				*A.t.* roots, cotyledons	PM, nucleus, cytosol		Krebs et al., 2012
YC4.6	ECFP-VENUS	58 nM 14.4 μM		*A.t.* pollen tubes	ER		Iwano et al., 2009 **Nagai et al., 2004**
D3cpv-KVK-SKL	ECFP-cpVENUS			*A.t.* leaves, guard cells	Peroxisomes	Ca^2+^	Costa et al., 2010 **Palmer et al., 2006**
D3cpv		0.6 μM		*A.t.* Roots, cotyledons	Tonoplast		Krebs et al., 2012
**pH SENSORS**							
pHluorin (ratiometric)	GFP	pKa 6.9		*N. tabacum,* pollen tubes	Cytosol		Certal et al., 2008; Michard et al., 2008 **Miesenbock et al., 1998**
				*A.t.* roots, *N. benthamiana*	Apoplast, cytosol	Fusicoccin salt, mannitol	Moseyko and Feldman, 2001; Plieth et al., 2001; Gao et al., 2004
PE-pHluorin PR-pHluorin	GFP	pKa 6.6	Ex 395/475 PE:Em 480/512 PR:Em 515	*A.t*. protoplasts PSB-D, PSB-L	Cytosol, vacuole, mitochondria, chloroplast endomembr. compartments		**Shen et al., 2013**
Pt-GFP	Pt-GFP	pKa 7.3 (7.8)	475/390 (ex) 508 (em)	*A.t.* roots, leaves guard cells	Cytosol		**Schulte et al., 2006**
GFP H148D	GFP	pKa 7.8		*A.t.* roots, root hairs	Cytosol	Gravitropism touch, barrier	Fasano et al., 2001; Monshausen et al., 2007, 2009, **Elsliger et al., 1999**
pHusion apo-pHusion	mRFP1-EGFP	pKa 5.8 (6.0)	EGFP: 488/507 mRFP1: 584/607	*A.t.* leaf mesophyll, roots	Cytosol, apoplast	IAA	**Gjetting et al., 2012**
**REDOX SENSORS**							
Cyt-RoGFP1 Cyt-GRX1- RoGFP1 c-RoGFP1/2 Per-RoGFP1 Pla-RoGFP2 MitRoGFP1+2	GFP	Mid point potential RoGFP1: 288 mV RoGFP2: 272 mV	RoGFP1: λ_max_ oxidized: 396 nm λ_max_ reduced: 468 nm	*A.t.* leaf discs, leaf epidermis, roots	Cytosol, peroxisomes, plastids, mitochondria	Dark, (age), inhibitors/abiotic, stress	Jiang et al., 2006; Schwarzlander et al., 2009; Rosenwasser et al., 2010 **Hanson et al., 2004**
RoGFP1 c-RoGFP1/2 Px-RoGFP2 Cp-RoGFP2 ER-RoGFP2			RoGFP2: λ_max_ oxidized: 398 nm λ_max_ reduced: 492 nm	*N. tabacum,* leaf epidermis, leaf discs	Cytosol, peroxisomes, plastids, ER	Inhibitors/abiotic stress	Schwarzlander et al., 2008, 2009
H_2_O_2_: HyPer HyPer-KSRM	cpYFP		Ex. 420/500 Em. 516	*N. tabacum* leaves, *A.t.* leaves pre-/post-bolting, guard cells	Cytosol, peroxisomes	Ca^2+^	Costa et al., 2010 **Belousov et al., 2006**
**OTHER SENSORS**							
Chloride: Clomeleon	ECFP-EYFP		ECFP: 434/477	*A.t.* roots	Cytosol	Salt stress, Mg^2+^, Ca^2+^, La^3+^, A9C (anion chan. block.)	Lorenzen et al., 2004; Plieth and Saleh, 2013, **Kuner and Augustine, 2000**
Glucose: FlipGlu600 μ, 2 m, 170 n	ECFP-EYFP	600 μM, 2 mM, 170 nM	EYFP: 514/427	*A.t.* silencing mutants and wildtype, roots, leaves	Cytosol	Glucose flux and levels	**Deuschle et al., 2006**; Chaudhuri et al., 2008, 2011
Sucrose: FlipSuc90 μ	ECFP-EYFP	88 μM		*A.t.* silencing mutants, roots	Cytosol		**Chaudhuri et al., 2008**
Glutamine: D157N	ECFP-Venus	6 mM (8.5 mM)	Venus:515/528	*A.t.* silencing mutants roots	Cytosol		**Yang et al., 2010**
PtdIns3P, 4P, (4,5)P_2_: YFP-2xFYVE mRFP-PH_FAPP1_ YFP-PH_FAPP1/PLC∂1_	EYFP/mRFP1		EYFP: 514/427 mRFP1: 584/607	Protoplasts (cowpea, BY2). *M. truncatula* roots, *A.t.* seedlings	Localization (PM, golgi, cell plate)	PPIs dynamics	Vermeer et al., 2006; Vermeer et al, 2009; van Leeuwen et al., 2007
Auxin: DII-VENUS	Venus		Venus: 515/528	*A.t.* leaves, roots	Nucleus	Gravitropism	**Brunoud et al., 2012**

*Details of the sensors: Dissociation constants, (Kd), pKa-values (of the pH sensors), with in vivo values in brackets, type of FP used, tissue, species, and potentially applied stimulus are given, as well as references to the original plant experiments. A.t. refers to Arabidopsis thaliana. References to Kd/pKa values and spectral properties of the sensors are shown in bold*.

Looking closer at these experiments, some obvious similarities are seen. It can be argued that successful experiments are often carried out in single cell systems, such as guard cells, root hairs and pollen tubes, where complex cell-to-cell communication is limited. These experiments are all studies of ion signaling, that can be directly correlated with a growth or turgor response, making them attractive experimental setups. Although this is a trend, indeed, several experiments have been successfully carried out in intact tissue, very often in roots, (Fasano et al., 2001; Rincon-Zachary et al., 2010; Monshausen et al., 2011; Gjetting et al., 2012) where autofluorescence is negligible and access is not hindered by the waxy cuticle. Sensors that were successfully used for physiological measurements in intact tissue were often developed specifically for use in plants (e.g., the auxin sensor, DII-Venus, and the apoplastic pH-sensor apo-pHusion). Secondly, it is noted that overall only few sensor platforms and targets were used, again reflecting the fact that many sensors are originally designed for mammalian purposes. Thirdly, sensors were most often expressed in the cytosol, which is the default expression if not specifically targeted to other compartments, and finally most experiments were carried out in Arabidopsis or tobacco, which may not be surprising, since these are easy to manipulate. These observations emphasize some specific challenges that have to be addressed in order to broaden the palette of successful sensor applications in plants.

## Plant-specific features that limit the applicability of genetically encoded fluorescent sensors

### Autofluorescence from chloroplasts and cell walls

Plants are complex, multicellular organisms to work with, and fluorescent probes do not always penetrate multiple cell layers, largely due to the barrier formed by the waxy cuticle and the cell wall. Therefore, genetically encoded sensors are ideally suited for plant cell imaging. However, in plants autofluorescence is a major challenge, particularly in photosynthesizing tissue (chlorophyll ex. 420–460 nm/em. 600–700 nm) and from the cell wall (various components are excited by UV to blue wavelengths, emitting mainly blue light), which can be addressed by the choice of fluorophores in sensor design, or may be circumvented, when lower photon counts/densities can be tolerated, by the use of bioluminescent proteins, such as Aequorin (Mehlmer et al., 2012), where excitation is caused by a chemical reaction instead of light, thus avoiding excitation of autofluorescence.

### Precautions for mounting procedures and studying externally applied chemical stimuli

Genetically encoded sensors as such are non-invasive, but their application to study cellular responses to chemical stimuli requires a perfusion setup and immobilization for microscopy, which can potentially harm the cells. The cuticle covering aerial tissues is an entrance barrier for many compounds, and sometimes even for the ligand itself, making *in vivo* calibrations difficult when using such sensors. Efficient immobilization methods ensure that no movement of the specimen takes place, while at the same time allowing for perfusion of the chemical stimulus and plant growth. It was, however, recently shown that the commonly used method to immobilize Arabidopsis tissue with a medical adhesive severely impairs cell viability of root cells (Gjetting et al., 2012), making alternative methods necessary. An alternative could be the newly developed root chip (Grossmann et al., 2011) or more simply mounting roots on agarose (Gjetting et al., 2012). Another common method used e.g., for cross-fixing pollen tubes on polylysine slides (Michard et al., 2008) was also shown to disrupt Arabidopsis root cells. In general, the act of handling living tissue under a microscope will inevitably cause disturbance of the tissue and induce various stress responses and tropisms. This of course affects live imaging methods of genetically encoded fluorescent sensors as well as other methods.

### Gene silencing may be caused by choice of promoter or tandem fluorescent proteins

Gene silencing has often been mentioned as a particular problem for plant expression of genetically encoded fluorescent biosensors, particularly when used in tandem repeats, or driven by the 35S promoter (Miyawaki et al., 1997; Deuschle et al., 2006; Krebs et al., 2012). This problem was solved in one case by replacing the 35S-promoter of viral origin with the plant-derived UBQ10 promoter in Arabidopsis (Krebs et al., 2012), or by expressing the sensor in transgene silencing mutants (Deuschle et al., 2006). The use of silencing mutants however, is not optimal, since their general growth pattern is changed, and may influence the measurements in unpredictable ways. In our lab, the 35S-promoter did not provoke inhibitory gene silencing when driving the expression of either FRET-based sensors or ratiometric pH sensors (Gjetting et al., 2012 and unpublished results). Transgene silencing in root tips and seedlings was reported to cause a reduction in fluorescence intensity and thus undetectable FRET changes after 10–15 days of growth (Deuschle et al., 2006; Chaudhuri et al., 2011). In contrast, we were able to monitor pH changes in leaves of 1–2 months old plants which were not subject to silencing (Gjetting et al., 2012).

### The apoplast

This plant-specific extracellular compartment plays a major role in transport regulation, but obviously only plant scientists are interested in developing tools to study its dynamics. Sensors for apoplastic measurements must deal with the low pH values, which are disruptive to many fluorescent proteins, and also be able to measure large differences in ion or solute concentration in the much less buffered apoplast. Apoplastic pH sensors have been used to measure salt stress (Schulte et al., 2006) and the effect of externally applied auxin (Gjetting et al., 2012), but the targeting of sensor protein to the apoplast results in accumulation in the ER, which should be taken into account when measuring the ratio. However, this accumulated protein could potentially be used as an internal pH reference or even as a tool to study pH in the endomembrane system as well. Another issue with apoplastic measurements relate to the structure of GFP in that an oxidizing environment, such as the cell wall and ER can impair proper folding of the fluorescent protein. The use of superfolder GFP (sfGFP) variants may in time be helpful in plants for solving this problem (Aronson et al., 2011).

## Improvement of sensor applications in plants

### Increasing sensor target range to include, e.g., hormones and kinases

There are many possibilities to expand the range of sensor targets in plants. Developing sensors for central, plant-specific signaling events, like hormone action or activity of plant-specific receptor-like kinases would be major landmarks. An example is a recently developed auxin sensor, which is a fusion of the YFP variant Venus to the Aux/IAA auxin-interaction domain DII (Brunoud et al., 2012), targeted to the nucleus. Using this sensor, auxin distribution was mapped during gravity sensing and lateral shoot formation in Arabidopsis. For mammalian cells, e.g., a variety of GFP-based biosensors exist for kinases, GTPases, phosphatidylinositols (PtdIns) (Kimber et al., 2002; Yoshizaki et al., 2006; Zhang and Allen, 2007). Such sensors (PtdIns) have only recently emerged in the plant community (Munnik and Nielsen, 2011), probably because plants use different signaling components that cannot be targeted by sensors developed for mammalian systems.

### pH measurements in acidic compartments

Sensors in plants have so far mainly been expressed in the cytosol, although several other compartments have also been explored (see Table 1). Indeed, targeted sensors are desirable, e.g., to study cell wall pH-dynamics (Gao et al., 2004; Gjetting et al., 2012). Sensor secretion to the apoplast involves accumulation of protein in transit in the endomembrane system, which is a problem to be considered carefully. This may be the reason that some researchers prefer pH-sensitive, small molecular weight fluorescent probes for surface pH measurements in Arabidopsis (Bibikova et al., 1998; Monshausen et al., 2011; Geilfus and Muhling, 2012). However, an apoplastic sensor, stably expressed in cells throughout the tissue, and not just the surface is preferable e.g., in roots to study details of the extracellular pH signature of gravitropic responses and auxin signaling (Swarup et al., 2005). The localization of pH sensors in the acidic compartment of the apoplast or vacuole is also hindered by the sensitivity of GFP to acidity (Tsien, 1998). The pH sensor *pt*GFP, derived from the Orange Seapen, *Ptilosarcus gurneyi* showed increased acid stability compared with avGFP derived pHluorins. PtGFP fluorescence could be fully restored after exposure to pH 3.5, and partially restored down to pH 2.5 and may therefore be more suitable for acidic measurements. In contrast, pHluorins were completely denatured at pH 3.5 (Schulte et al., 2006). Recently, a pHluorin-derived sensor, based on a solubility-modified GFP (sm-GFP) was targeted to the vacuole, and to other endomembrane compartments and used to determine pH of the different compartments (Shen et al., 2013).

### Variety of FPs and technology

Expanding the variety of sensor fluorescent proteins, e.g., by the development of different FRET donor/acceptors would facilitate the study of several ions/metabolites simultaneously, e.g., the commonly linked signaling cascade of intracellular calcium/apoplastic pH, as well as same ion fluxes in several compartments or complex protein-protein interactions. Multiplexed FRET (Piljic and Schultz, 2008) and fluorescence lifetime imaging (FLIM)-FRET (Grant et al., 2008) are becoming more feasible as the variety of spectral variants increases. In *N. benthamiana* leaves a FRET-FLIM assay was used to detect known protein interactions using a FRET pair of the GFP variant TSapphire as donor, and mOrange as acceptor (Bayle et al., 2008). A similar approach was used to detect a flavonoid metabolon in Arabidopsis protoplasts (Crosby et al., 2011). These examples are not using genetically encoded sensors as such, and are based on transient expression in single cell systems, but further illustrate the possibilities of using fluorescent protein technology in plants and may be useful for sensor construction at a later stage.

### Identification of new genes and gene function

Genetically encoded sensors may also be used to identify the role of genes of unknown function. A new class of glucose efflux transporters, SWEETs was identified by FRET-based glucose sensors (Chen et al., 2010), and repeated with a sucrose sensor, identifying a subclade of SWEET efflux transporters involved in sucrose transport, indicating a role in phloem loading (Chen et al., 2011). Another promising sensor application known from animal systems, addressed the functional identification of unknown signaling components. This idea was elegantly adapted to Arabidopsis, where the luminescent calcium sensor Aequorin was used to identify an extracellular signaling peptide, AtRALF1, (rapid alkalinization factor) by its ability to induce a cytosolic Ca^2+^-increase (Haruta et al., 2008). The effect of this peptide was subsequently analysed in detail in Arabidopsis roots expressing the Cameleon sensor YC3.6.

## Concluding remarks

The use of genetically encoded sensors in plants faces some specific challenges not shared with the mammalian world, which need to be addressed by plant scientists. Nevertheless, the continuous development and refinement of fluorescent proteins, sensor design and bioimaging techniques make genetically encoded sensors very promising tools for elucidating metabolic networks and signaling events in plant cells in the future.

### Conflict of interest statement

The authors declare that the research was conducted in the absence of any commercial or financial relationships that could be construed as a potential conflict of interest.

## References

[B1] Allen G. J. Chu S. P. Harrington C. L. Schumacher K. Hoffmann T. Tang Y. Y. (2001). A defined range of guard cell calcium oscillation parameters encodes stomatal movements. Nature 411, 1053–1057 10.1038/35082575 11429606

[B2] Allen G. J. Chu S. P. Schumacher K. Shimazaki C. T. Vafeados D. Kemper A. (2000). Alteration of stimulus-specific guard cell calcium oscillations and stomatal closing in Arabidopsis *det3* mutant. Science 289, 2338–2342 10.1126/science.289.5488.2338 11009417

[B3] Allen G. J. Kuchitsu K. Chu S. P. Murata Y. Schroeder J. I. (1999a). Arabidopsis *abi1-1* and *abi2-1* phosphatase mutations reduce abscisic acid-induced cytoplasmic calcium rises in guard cells. Plant Cell 11, 1785–1798 10488243 10.1105/tpc.11.9.1785 PMC144302

[B4] Allen G. J. Kwak J. M. Chu S. P. Llopis J. Tsien R. Y. Harper J. F. (1999b). Cameleon calcium indicator reports cytoplasmic calcium dynamics in Arabidopsis guard cells. Plant J. 19, 735–747 10.1046/j.1365-313x.1999.00574.x 10571859

[B5] Allen G. J. Murata Y. Chu S. P. Nafisi M. Schroeder J. I. (2002). Hypersensitivity of abscisic acid-induced cytosolic calcium increases in the Arabidopsis farnesyltransferase mutant *era1-2*. Plant Cell 14, 1649–1662 10.1105/tpc.010448 12119381 PMC150713

[B6] Aronson D. E. Costantini L. M. Snapp E. L. (2011). Superfolder GFP is fluorescent in oxidizing environments when targeted via the Sec translocon. Traffic 12, 543–548 10.1111/j.1600-0854.2011.01168.x 21255213 PMC3079558

[B7] Bayle V. Nussaume L. Bhat R. A. (2008). Combination of novel green fluorescent protein mutant TSapphire and DsRed variant mOrange to set up a versatile in planta FRET-FLIM assay. Plant Physiol. 148, 51–60 10.1104/pp.108.117358 18621983 PMC2528103

[B8] Belousov V. V. Fradkov A. F. Lukyanov K. A. Staroverov D. B. Shakhbazov K. S. Terskikh A. V. (2006). Genetically encoded fluorescent indicator for intracellular hydrogen peroxide. Nat. Methods 3, 281–286 10.1038/nmeth866 16554833

[B9] Bibikova T. N. Jacob T. Dahse I. Gilroy S. (1998). Localized changes in apoplastic and cytoplasmic pH are associated with root hair development in Arabidopsis thaliana. Development 125, 2925–2934 9655814 10.1242/dev.125.15.2925

[B10] Brunoud G. Wells D. M. Oliva M. Larrieu A. Mirabet V. Burrow A. H. (2012). A novel sensor to map auxin response and distribution at high spatio-temporal resolution. Nature 482, 103–106 10.1038/nature10791 22246322

[B11] Capoen W. Sun J. Wysham D. Otegui M. S. Venkateshwaran M. Hirsch S. (2011). Nuclear membranes control symbiotic calcium signaling of legumes. Proc. Natl. Acad. Sci. U.S.A. 108, 14348–14353 10.1073/pnas.1107912108 21825141 PMC3161518

[B12] Certal A. C. Almeida R. B. Carvalho L. M. Wong E. Moreno N. Michard E. (2008). Exclusion of a proton ATPase from the apical membrane is associated with cell polarity and tip growth in *Nicotiana tabacum* pollen tubes. Plant Cell 20, 614–634 10.1105/tpc.106.047423 18364468 PMC2329945

[B13] Chaudhuri B. Hormann F. Frommer W. B. (2011). Dynamic imaging of glucose flux impedance using FRET sensors in wild-type Arabidopsis plants. J. Exp. Bot. 62, 2411–2417 10.1093/jxb/erq444 21266495

[B14] Chaudhuri B. Hormann F. Lalonde S. Brady S. M. Orlando D. A. Benfey P. (2008). Protonophore- and pH-insensitive glucose and sucrose accumulation detected by FRET nanosensors in Arabidopsis root tips. Plant J. 56, 948–962 10.1111/j.1365-313X.2008.03652.x 18702670 PMC2752219

[B15] Chen L. Q. Hou B. H. Lalonde S. Takanaga H. Hartung M. L. Qu X. Q. (2010). Sugar transporters for intercellular exchange and nutrition of pathogens. Nature 468, 527–532 10.1038/nature09606 21107422 PMC3000469

[B16] Chen L. Q. Qu X. Q. Hou B. H. Sosso D. Osorio S. Fernie A. R. (2011). Sucrose efflux mediated by SWEET proteins as a key step for phloem transport. Science 335, 207–211 10.1126/science.1213351 22157085

[B17] Choi W. G. Swanson S. J. Gilroy S. (2012). High-resolution imaging of Ca^2+^, redox status, ROS and pH using GFP biosensors. Plant J. 70, 118–128 10.1111/j.1365-313X.2012.04917.x 22449047

[B18] Chudakov D. M. Matz M. V. Lukyanov S. Lukyanov K. A. (2010). Fluorescent proteins and their applications in imaging living cells and tissues. Physiol. Rev. 90, 1103–1163 10.1152/physrev.00038.2009 20664080

[B19] Costa A. Drago I. Behera S. Zottini M. Pizzo P. Schroeder J. I. (2010). H2O2 in plant peroxisomes: an *in vivo* analysis uncovers a Ca^2+^-dependent scavenging system. Plant J. 62, 760–772 10.1111/j.1365-313X.2010.04190.x 20230493 PMC2884081

[B20] Crosby K. C. Pietraszewska-Bogiel A. Gadella T. W. Jr. Winkel B. S. (2011). Forster resonance energy transfer demonstrates a flavonoid metabolon in living plant cells that displays competitive interactions between enzymes. FEBS Lett. 585, 2193–2198 10.1016/j.febslet.2011.05.066 21669202

[B21] Deuschle K. Chaudhuri B. Okumoto S. Lager I. Lalonde S. Frommer W. B. (2006). Rapid metabolism of glucose detected with FRET glucose nanosensors in epidermal cells and intact roots of Arabidopsis RNA-silencing mutants. Plant Cell 18, 2314–2325 10.1105/tpc.106.044073 16935985 PMC1560921

[B22] Dixit R. Cyr R. Gilroy S. (2006). Using intrinsically fluorescent proteins for plant cell imaging. Plant J. 45, 599–615 10.1111/j.1365-313X.2006.02658.x 16441351

[B23] Ehrhardt D. W. Frommer W. B. (2012). New technologies for 21st century plant science. Plant Cell 24, 374–394 10.1105/tpc.111.093302 22366161 PMC3315222

[B24] Elsliger M. A. Wachter R. M. Hanson G. T. Kallio K. Remington S. J. (1999). Structural and spectral response of green fluorescent protein variants to changes in pH. Biochemistry 38, 5296–5301 10.1021/bi9902182 10220315

[B25] Fasano J. M. Swanson S. J. Blancaflor E. B. Dowd P. E. Kao T. H. Gilroy S. (2001). Changes in root cap pH are required for the gravity response of the Arabidopsis root. Plant Cell 13, 907–921 11283344 10.1105/tpc.13.4.907 PMC135544

[B26] Fehr M. Ehrhardt D. W. Lalonde S. Frommer W. B. (2004). Minimally invasive dynamic imaging of ions and metabolites in living cells. Curr. Opin. Plant Biol. 7, 345–351 10.1016/j.pbi.2004.03.015 15134757

[B27] Frommer W. B. Davidson M. W. Campbell R. E. (2009). Genetically encoded biosensors based on engineered fluorescent proteins. Chem. Soc. Rev. 38, 2833–2841 10.1039/b907749a 19771330 PMC3000468

[B28] Gao D. Knight M. R. Trewavas A. J. Sattelmacher B. Plieth C. (2004). Self-reporting Arabidopsis expressing pH and Ca^2+^ indicators unveil ion dynamics in the cytoplasm and in the apoplast under abiotic stress. Plant Physiol. 134, 898–908 10.1104/pp.103.032508 15020753 PMC389913

[B29] Geilfus C. M. Muhling K. H. (2012). Transient alkalinization in the leaf apoplast of *Vicia faba L.* depends on NaCl stress intensity: an *in situ* ratio imaging study. Plant Cell Environ. 35, 578–587 10.1111/j.1365-3040.2011.02437.x 21954856

[B30] Gjetting K. S. Ytting C. K. Schulz A. Fuglsang A. T. (2012). Live imaging of intra- and extracellular pH in plants using pHusion, a novel genetically encoded biosensor. J. Exp. Bot. 63, 3207–3218 10.1093/jxb/ers040 22407646 PMC3350929

[B31] Grant D. M. Zhang W. McGhee E. J. Bunney T. D. Talbot C. B. Kumar S. (2008). Multiplexed FRET to image multiple signaling events in live cells. Biophys. J. 95, L69–L71 10.1529/biophysj.108.139204 18757561 PMC2576366

[B32] Grossmann G. Guo W. J. Ehrhardt D. W. Frommer W. B. Sit R. V. Quake S. R. (2011). The RootChip: an integrated microfluidic chip for plant science. Plant Cell 23, 4234–4240 10.1105/tpc.111.092577 22186371 PMC3269862

[B33] Hanson G. T. Aggeler R. Oglesbee D. Cannon M. Capaldi R. A. Tsien R. Y. (2004). Investigating mitochondrial redox potential with redox-sensitive green fluorescent protein indicators. J. Biol. Chem. 279, 13044–13053 10.1074/jbc.M312846200 14722062

[B34] Haruta M. Monshausen G. Gilroy S. Sussman M. R. (2008). A cytoplasmic Ca^2+^ functional assay for identifying and purifying endogenous cell signaling peptides in Arabidopsis seedlings: identification of AtRALF1 peptide. Biochemistry 47, 6311–6321 10.1021/bi8001488 18494498

[B35] Hugouvieux V. Kwak J. M. Schroeder J. I. (2001). An mRNA cap binding protein, ABH1, modulates early abscisic acid signal transduction in Arabidopsis. Cell 106, 477–487 10.1016/S0092-8674(01)00460-3 11525733

[B36] Islam M. M. Hossain M. A. Jannat R. Munemasa S. Nakamura Y. Mori I. C. (2010). Cytosolic alkalization and cytosolic calcium oscillation in arabidopsis guard cells response to ABA and MeJA. Plant Cell Physiol. 51, 1721–1730 10.1093/pcp/pcq131 20739306

[B37] Iwano M. Entani T. Shiba H. Kakita M. Nagai T. Mizuno H. (2009). Fine-tuning of the cytoplasmic Ca^2+^ concentration is essential for pollen tube growth. Plant Physiol. 150, 1322–1334 10.1104/pp.109.139329 19474213 PMC2705041

[B38] Iwano M. Shiba H. Miwa T. Che F. S. Takayama S. Nagai T. (2004). Ca^2+^ dynamics in a pollen grain and papilla cell during pollination of Arabidopsis. Plant Physiol. 136, 3562–3571 10.1104/pp.104.046961 15489279 PMC527155

[B39] Jiang K. Schwarzer C. Lally E. Zhang S. Ruzin S. Machen T. (2006). Expression and characterization of a redox-sensing green fluorescent protein (reduction-oxidation-sensitive green fluorescent protein) in Arabidopsis. Plant Physiol. 141, 397–403 10.1104/pp.106.078246 16760494 PMC1475439

[B40] Kimber W. A. Trinkle-Mulcahy L. Cheung P. C. Deak M. Marsden L. J. Kieloch A. (2002). Evidence that the tandem-pleckstrin-homology-domain-containing protein TAPP1 interacts with Ptd(3, 4)P_2_ and the multi-PDZ-domain-containing protein MUPP1 *in vivo*. Biochem. J. 361, 525–536 10.1042/0264-6021:3610525 11802782 PMC1222335

[B41] Klusener B. Young J. J. Murata Y. Allen G. J. Mori I. C. Hugouvieux V. (2002). Convergence of calcium signaling pathways of pathogenic elicitors and abscisic acid in Arabidopsis guard cells. Plant Physiol. 130, 2152–2163 10.1104/pp.012187 12481099 PMC166727

[B42] Kosuta S. Hazledine S. Sun J. Miwa H. Morris R. J. Downie J. A. (2008). Differential and chaotic calcium signatures in the symbiosis signaling pathway of legumes. Proc. Natl. Acad. Sci. U.S.A. 105, 9823–9828 10.1073/pnas.0803499105 18606999 PMC2474534

[B43] Krebs M. Held K. Binder A. Hashimoto K. Den Herder G. Parniske M. (2012). FRET-based genetically encoded sensors allow high-resolution live cell imaging of Ca^2+^ dynamics. Plant J. 69, 181–192 10.1111/j.1365-313X.2011.04780.x 21910770

[B44] Kuner T. Augustine G. J. (2000). A genetically encoded ratiometric indicator for chloride: capturing chloride transients in cultured hippocampal neurons. Neuron 27, 447–459 10.1016/S0896-6273(00)00056-8 11055428

[B45] Lalonde S. Ehrhardt D. W. Frommer W. B. (2005). Shining light on signaling and metabolic networks by genetically encoded biosensors. Curr. Opin. Plant Biol. 8, 574–581 10.1016/j.pbi.2005.09.015 16188489 PMC2740940

[B46] Lorenzen I. Aberle T. Plieth C. (2004). Salt stress-induced chloride flux: a study using transgenic Arabidopsis expressing a fluorescent anion probe. Plant J. 38, 539–544 10.1111/j.0960-7412.2004.02053.x 15086798

[B47] Mehlmer N. Parvin N. Hurst C. H. Knight M. R. Teige M. Vothknecht U. C. (2012). A toolset of aequorin expression vectors for in planta studies of subcellular calcium concentrations in *Arabidopsis thaliana*. J. Exp. Bot. 63, 1751–1761 10.1093/jxb/err406 22213817 PMC3971373

[B48] Mehta S. Zhang J. (2011). Reporting from the field: genetically encoded fluorescent reporters uncover signaling dynamics in living biological systems. Annu. Rev. Biochem. 80, 375–401 10.1146/annurev-biochem-060409-093259 21495849 PMC4384825

[B49] Michard E. Dias P. Feijo J. (2008). Tobacco pollen tubes as cellular models for ion dynamics: improved spatial and temporal resolution of extracellular flux and free cytosolic concentration of calcium and protons using pHluorin and YC3.1 Cameleon. Sex Plant Reprod. 21, 169–181 10.1007/s00497-008-0076-x

[B50] Michard E. Lima P. T. Borges F. Silva A. C. Portes M. T. Carvalho J. E. (2011). Glutamate receptor-like genes form Ca^2+^ channels in pollen tubes and are regulated by pistil D-serine. Science 332, 434–437 10.1126/science.1201101 21415319

[B51] Miesenbock G. De Angelis D. A. Rothman J. E. (1998). Visualizing secretion and synaptic transmission with pH-sensitive green fluorescent proteins. Nature 394, 192–195 10.1038/28190 9671304

[B52] Miwa H. Sun J. Oldroyd G. E. Downie J. A. (2006a). Analysis of Nod-factor-induced calcium signaling in root hairs of symbiotically defective mutants of *Lotus japonicus*. Mol. Plant Microbe Interact. 19, 914–923 10.1094/MPMI-19-0914 16903357

[B53] Miwa H. Sun J. Oldroyd G. E. Downie J. A. (2006b). Analysis of calcium spiking using a Cameleon calcium sensor reveals that nodulation gene expression is regulated by calcium spike number and the developmental status of the cell. Plant J. 48, 883–894 10.1111/j.1365-313X.2006.02926.x 17227545

[B54] Miyawaki A. (2011). Proteins on the move: insights gained from fluorescent protein technologies. Nat. Rev. Mol. Cell Biol. 12, 656–668 10.1038/nrm3199 21941275

[B55] Miyawaki A. Griesbeck O. Heim R. Tsien R. Y. (1999). Dynamic and quantitative Ca^2+^ measurements using improved Cameleons. Proc. Natl. Acad. Sci. U.S.A. 96, 2135–2140 10.1073/pnas.96.5.2135 10051607 PMC26749

[B56] Miyawaki A. Llopis J. Heim R. McCaffery J. M. Adams J. A. Ikura M. (1997). Fluorescent indicators for Ca^2+^ based on green fluorescent proteins and calmodulin. Nature 388, 882–887 10.1038/42264 9278050

[B57] Monshausen G. B. Bibikova T. N. Messerli M. A. Shi C Gilroy S. (2007). Oscillations in extracellular pH and reactive oxygen species modulate tip growth of Arabidopsis root hairs. Proc. Natl. Acad. Sci. U.S.A. 104, 20996–21001 10.1073/pnas.0708586104 18079291 PMC2409255

[B58] Monshausen G. B. Bibikova T. N. Weisenseel M. H. Gilroy S. (2009). Ca^2+^ regulates reactive oxygen species production and pH during mechanosensing in Arabidopsis roots. Plant Cell 21, 2341–2356 10.1105/tpc.109.068395 19654264 PMC2751959

[B59] Monshausen G. B. Messerli M. A. Gilroy S. (2008). Imaging of the Yellow Cameleon 3.6 indicator reveals that elevations in cytosolic Ca^2+^ follow oscillating increases in growth in root hairs of Arabidopsis. Plant Physiol. 147, 1690–1698 10.1104/pp.108.123638 18583529 PMC2492656

[B60] Monshausen G. B. Miller N. D. Murphy A. S. Gilroy S. (2011). Dynamics of auxin-dependent Ca^2+^ and pH signaling in root growth revealed by integrating high-resolution imaging with automated computer vision-based analysis. Plant J. 65, 309–318 10.1111/j.1365-313X.2010.04423.x 21223394

[B61] Moseyko N. Feldman L. J. (2001). Expression of pH-sensitive green fluorescent protein in *Arabidopsis thaliana*. Plant Cell Environ. 24, 557–563 10.1046/j.1365-3040.2001.00703.x 11706851

[B62] Munemasa S. Hossain M. A. Nakamura Y. Mori I. C. Murata Y. (2011). The Arabidopsis calcium-dependent protein kinase, CPK6, functions as a positive regulator of methyl jasmonate signaling in guard cells. Plant Physiol. 155, 553–561 10.1104/pp.110.162750 20978156 PMC3075756

[B63] Munnik T. Nielsen E. (2011). Green light for polyphosphoinositide signals in plants. Curr. Opin. Plant Biol. 14, 489–497 10.1016/j.pbi.2011.06.007 21775194

[B64] Nagai T. Yamada S. Tominaga T. Ichikawa M. Miyawaki A. (2004). Expanded dynamic range of fluorescent indicators for Ca^2+^ by circularly permuted yellow fluorescent proteins. Proc. Natl. Acad. Sci. U.S.A. 101, 10554–10559 10.1073/pnas.0400417101 15247428 PMC490022

[B65] Newman R. H. Fosbrink M. D. Zhang J. (2011). Genetically encodable fluorescent biosensors for tracking signaling dynamics in living cells. Chem. Rev. 111, 3614–3666 10.1021/cr100002u 21456512 PMC3092831

[B66] Okumoto S. (2010). Imaging approach for monitoring cellular metabolites and ions using genetically encoded biosensors. Curr. Opin. Biotechnol. 21, 45–54 10.1016/j.copbio.2010.01.009 20167470 PMC2843770

[B67] Okumoto S. (2012). Quantitative imaging using genetically encoded sensors for small molecules in plants. Plant J. 70, 108–117 10.1111/j.1365-313X.2012.04910.x 22449046

[B68] Okumoto S. Jones A. Frommer W. B. (2012). Quantitative imaging with fluorescent biosensors: advanced tools for spatiotemporal analysis of biodynamics in cells. Annu. Rev. Plant Biol. 63, 663–706 10.1146/annurev-arplant-042110-103745 22404462

[B69] Palmer A. E. Giacomello M. Kortemme T. Hires S. A. Lev-Ram V. Baker D. (2006). Ca^2+^ indicators based on computationally redesigned calmodulin-peptide pairs. Chem. Biol. 13, 521–530 10.1016/j.chembiol.2006.03.007 16720273

[B70] Palmer A. E. Qin Y. Park J. G. McCombs J. E. (2011). Design and application of genetically encoded biosensors. Trends Biotechnol. 29, 144–152 10.1016/j.tibtech.2010.12.004 21251723 PMC3433949

[B71] Piljic A. Schultz C. (2008). Simultaneous recording of multiple cellular events by FRET. ACS Chem. Biol. 3, 156–160 10.1021/cb700247q 18355004

[B72] Plieth C. Saleh L. (2013). A9C sensitive Cl(-)-accumulation in *A. thaliana* root cells during salt stress is controlled by internal and external calcium. Plant Signal. Behav. [Epub ahead of print]. 23603974 10.4161/psb.24259 PMC3925454

[B73] Plieth C. Sattelmacher B. Trewavas A. Hansen U. Knight M. (2001). Engineering plants expressiong calcium and pH indicators in the cytoplasm and the apoplast. Plant Nutri. 92, 252–253 10.1007/0-306-47624-X_121

[B74] Rincon-Zachary M. Teaster N. D. Sparks J. A. Valster A. H. Motes C. M. Blancaflor E. B. (2010). Fluorescence resonance energy transfer-sensitized emission of yellow Cameleon 3.60 reveals root zone-specific calcium signatures in Arabidopsis in response to aluminum and other trivalent cations. Plant Physiol. 152, 1442–1458 10.1104/pp.109.147256 20053711 PMC2832233

[B75] Rosenwasser S. Rot I. Meyer A. J. Feldman L. Jiang K. Friedman H. (2010). A fluorometer-based method for monitoring oxidation of redox-sensitive GFP (roGFP) during development and extended dark stress. Physiol. Plant 138, 493–502 10.1111/j.1399-3054.2009.01334.x 20051029

[B76] Schulte A. Lorenzen I. Bottcher M. Plieth C. (2006). A novel fluorescent pH probe for expression in plants. Plant Methods 2, 7 10.1186/1746-4811-2-7 16600023 PMC1475855

[B77] Schwarzlander M. Fricker M. D. Muller C. Marty L. Brach T. Novak J. (2008). Confocal imaging of glutathione redox potential in living plant cells. J. Microsc. 231, 299–316 10.1111/j.1365-2818.2008.02030.x 18778428

[B78] Schwarzlander M. Fricker M. D. Sweetlove L. J. (2009). Monitoring the *in vivo* redox state of plant mitochondria: effect of respiratory inhibitors, abiotic stress and assessment of recovery from oxidative challenge. Biochim. Biophys. Acta 1787, 468–475 10.1016/j.bbabio.2009.01.020 19366606

[B79] Shen J. Zeng Y. Zhuang X. Sun L. Yao X. Pimpl P. (2013). Organelle pH in the Arabidopsis endomembrane system. Mol. Plant. [Epub ahead of print]. 10.1093/mp/sst079 23702593

[B80] Sieberer B. J. Chabaud M. Timmers A. C. Monin A. Fournier J. Barker D. G. (2009). A nuclear-targeted Cameleon demonstrates intranuclear Ca^2+^ spiking in Medicago truncatula root hairs in response to rhizobial nodulation factors. Plant Physiol. 151, 1197–1206 10.1104/pp.109.142851 19700563 PMC2773104

[B81] Siegel R. S. Xue S. Murata Y. Yang Y. Nishimura N. Wang A. (2009). Calcium elevation-dependent and attenuated resting calcium-dependent abscisic acid induction of stomatal closure and abscisic acid-induced enhancement of calcium sensitivities of S-type anion and inward-rectifying K channels in Arabidopsis guard cells. Plant J. 59, 207–220 10.1111/j.1365-313X.2009.03872.x 19302418 PMC2827207

[B82] Sun J. Miwa H. Downie J. A. Oldroyd G. E. (2007). Mastoparan activates calcium spiking analogous to Nod factor-induced responses in Medicago truncatula root hair cells. Plant Physiol. 144, 695–702 10.1104/pp.106.093294 17322338 PMC1914167

[B83] Swanson S. J. Choi W. G. Chanoca A. Gilroy S. (2011). *In vivo* imaging of Ca^2+^, pH, and reactive oxygen species using fluorescent probes in plants. Annu. Rev. Plant Biol. 62, 273–297 10.1146/annurev-arplant-042110-103832 21370977

[B84] Swarup R. Kramer E. M. Perry P. Knox K. Leyser H. M. Haseloff J. (2005). Root gravitropism requires lateral root cap and epidermal cells for transport and response to a mobile auxin signal. Nat. Cell Biol. 7, 1057–1065 10.1038/ncb1316 16244669

[B85] Tsien R. Y. (1998). The green fluorescent protein. Annu. Rev. Biochem. 67, 509–544 10.1146/annurev.biochem.67.1.509 9759496

[B86] VanEngelenburg S. B. Palmer A. E. (2008). Fluorescent biosensors of protein function. Curr. Opin. Chem. Biol. 12, 60–65 10.1016/j.cbpa.2008.01.020 18282482

[B87] van Leeuwen W. Vermeer J. E. Gadella T. W. Jr. Munnik T. (2007). Visualization of phosphatidylinositol 4, 5-bisphosphate in the plasma membrane of suspension-cultured tobacco BY-2 cells and whole Arabidopsis seedlings. Plant J. 52, 1014–1026 10.1111/j.1365-313X.2007.03292.x 17908156

[B88] Vermeer J. E. Thole J. M. Goedhart J. Nielsen E. Munnik T. Gadella T. W. Jr. (2009). Imaging phosphatidylinositol 4-phosphate dynamics in living plant cells. Plant J. 57, 356–372 10.1111/j.1365-313X.2008.03679.x 18785997

[B89] Vermeer J. E. van Leeuwen W. Tobena-Santamaria R. Laxalt A. M. Jones D. R. Divecha N. (2006). Visualization of PtdIns3P dynamics in living plant cells. Plant J. 47, 687–700 10.1111/j.1365-313X.2006.02830.x 16856980

[B90] Watahiki M. K. Trewavas A. J. Parton R. M. (2004). Fluctuations in the pollen tube tip-focused calcium gradient are not reflected in nuclear calcium level: a comparative analysis using recombinant yellow Cameleon calcium reporter. Sex. Plant Reprod. 17, 125–130 10.1007/s00497-004-0224-x

[B91] Weinl S. Held K. Schlucking K. Steinhorst L. Kuhlgert S. Hippler M. (2008). A plastid protein crucial for Ca^2+^-regulated stomatal responses. New Phytol. 179, 675–686 10.1111/j.1469-8137.2008.02492.x 18507772

[B92] Yang H. Bogner M. Stierhof Y. D. Ludewig U. (2010). H-independent glutamine transport in plant root tips. PLoS ONE 5:e8917 10.1371/journal.pone.0008917 20111724 PMC2811748

[B93] Yoshizaki H. Aoki K. Nakamura T. Matsuda M. (2006). Regulation of RalA GTPase by phosphatidylinositol 3-kinase as visualized by FRET probes. Biochem. Soc. Trans. 34, 851–854 10.1042/BST0340851 17052213

[B94] Young J. J. Mehta S. Israelsson M. Godoski J. Grill E. Schroeder J. I. (2006). CO_2_ signaling in guard cells: calcium sensitivity response modulation, a Ca^2+^-independent phase, and CO(2) insensitivity of the gca2 mutant. Proc. Natl. Acad. Sci. U.S.A. 103, 7506–7511 10.1073/pnas.0602225103 16651523 PMC1464368

[B95] Zhang J. Allen M. D. (2007). FRET-based biosensors for protein kinases: illuminating the kinome. Mol. Biosyst. 3, 759–765 10.1039/b706628g 17940658

